# Etiological and clinical characteristics of central diabetes insipidus in children: a single center experience

**DOI:** 10.1186/s13633-016-0021-y

**Published:** 2016-02-11

**Authors:** Janel D. Hunter, Ali S. Calikoglu

**Affiliations:** Division of Pediatric Endocrinology, University of North Carolina at Chapel Hill, Campus Box #7039, Medical School Wing E, Chapel Hill, NC 27599 USA

**Keywords:** Diabetes insipidus, Hypopituitarism, Vasopressin, Magnetic resonance imaging

## Abstract

**Background:**

Central diabetes insipidus (CDI) results from a number of conditions affecting the hypothalamic-neurohypophyseal system to cause vasopressin deficiency. Diagnosis of CDI is challenging, and clinical data and guidelines for management are lacking. We aim to characterize clinical and radiological characteristics of a cohort of pediatric patients with CDI.

**Methods:**

A chart review of 35 patients with CDI followed at North Carolina Children’s Hospital from 2000 to 2015 was undertaken. The frequencies of specific etiologies of CDI and characteristic magnetic resonance imaging (MRI) findings were determined. The presence of additional hormone deficiencies at diagnosis and later in the disease course was ascertained. Patient characteristics and management strategies were evaluated.

**Results:**

The cohort included 14 female and 21 male patients with a median age of 4.7 years (range, less than 1 month to 16 years) at diagnosis. Median duration of follow-up was 5 years (range, 2 months to 16 years). The cause of CDI was intracranial mass in 13 patients (37.2 %), septo-optic dysplasia in 9 patients (25.7 %), holoprosencephaly in 5 patients (14.2 %), Langerhans cell histiocytosis in 3 patients (8.6 %), isolated pituitary hypoplasia in 2 patients (5.7 %), and encephalocele in 1 patient (2.9 %). Patients were symptomatic for a mean of 6.3 months (range, less than 1 month to 36 months) prior to diagnosis of CDI. Growth hormone (GH), thyrotropin (TSH), adrenocorticotropic hormone (ACTH), and gonadotropin deficiencies were present at diagnosis in 34, 23, 23, and 6 % of patients, respectively. GH, TSH, ACTH, and gonadotropin deficiencies were diagnosed during follow-up in 23, 40, 37, and 14 % of patients, respectively. In patients with structural CNS abnormalities, development of additional hormone deficiencies occurred anywhere from 2 months to 13 years after the time of initial presentation.

**Conclusions:**

All patients in our cohort had an underlying organic etiology for CDI, with intracranial masses and CNS malformations being most common. Therefore, MRI of the brain is indicated in all pediatric patients with CDI. Other pituitary hormone deficiencies should be investigated at diagnosis as well as during follow-up.

## Background

Central diabetes insipidus (CDI) is characterized by polyuria, polydipsia, and the inability to concentrate urine as a result of arginine vasopressin deficiency. Maintenance of normal tonicity of extracellular fluids is vital to cellular function and is regulated by the complex interaction of fluid intake, vasopressin secretion, and urine output. Dysfunction of the system that maintains water homeostasis may result in life-threatening hypernatremia, seizures, dehydration, and failure to thrive. Vasopressin is synthesized by neurons of the hypothalamic paraventricular and supraoptic nuclei and secreted by the posterior pituitary gland [1]. Therefore, malformation or damage to midline cerebral and cranial structures may result in the absence of vasopressin production or secretion. Known etiologies of CDI include central nervous system (CNS) tumors, post-neurosurgical or accidental trauma, autoimmune disease, or infiltrative diseases. Genetic mutations resulting in abnormal synthesis of vasopressin precursor hormones may occur rarely [2]. In a large Danish study in 2014, the annual incidence of CDI overall was 3 to 4 patients per 100,000 with an incidence of 2 cases of congenital CDI per 100,000 infants [3].

**Fig. 1:**
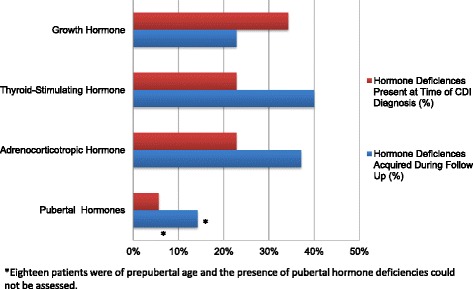
Concurrent Hormone Deficiencies at Central Diabetes Insipidus Diagnosis and Acquired During Follow Up

CDI presents a number of diagnostic and therapeutic challenges. The diagnosis may be delayed during evaluation for more common etiologies of polydipsia and polyuria, and often requires formal water deprivation testing [1]. After the diagnosis of CDI is confirmed, further work-up to determine the etiology of CDI and to assess for additional pituitary hormone deficiencies is necessary; however, clear guidelines for which laboratory and imaging studies to obtain and at what intervals to repeat these investigations are lacking.

The mainstay of therapy for CDI is desmopressin (DDAVP), a synthetic analog of arginine vasopressin with an extended duration of action; however, education of patients and caregivers regarding the safe use of DDAVP is essential to minimize risks of water-intoxication and hyponatremia. Risks of DDAVP-associated hyponatremia are increased in neonates, in patients without an intact thirst mechanism, and in patients requiring high-volume intravenous fluid hydration for chemotherapy. Given the number of diagnostic and therapeutic challenges associated with CDI, we performed a retrospective chart review of a cohort of pediatric patients with CDI followed at the North Carolina Children’s Hospital (NCCH) in order to describe our experience and to contribute further to generalizable knowledge about the diagnosis and management of CDI.

## Methods

The study was approved by the University of North Carolina Institutional Review Board, and conducted in compliance with the Helsinki Declaration. The NCCH electronic medical record was reviewed in order to identify all patients with CDI followed in pediatric endocrinology clinic. All patients that have been evaluated for management or diagnosis of CDI in the past year were included. Extracted data included patient characteristics (age, gender, race), duration of clinical symptoms prior to diagnosis in those presenting with CDI, pituitary hormone laboratory evaluation, family history, neuroimaging study results, and pathology findings. Management strategies were also reviewed.

The frequencies of various etiologies of CDI as well as characteristic magnetic resonance imaging (MRI) findings were determined. CDI was diagnosed either by water deprivation testing or by the presence of concurrent polyuria, hypernatremia, elevated serum osmolality, low urine osmolality, and low urine specific gravity. The prevalence of additional anterior pituitary hormone deficiencies at each patient’s presentation and the incidence of hormone deficiencies acquired later in the disease course were also evaluated. The presence of a hormone deficiency was confirmed by laboratory evidence of insufficient hormone production.

## Results

The cohort included 21 male patients and 14 female patients whose median age at diagnosis of CDI was 4.8 years (range, less than 1 month to 16 years). In the patients with CDI at their initial presentation, mean duration of symptoms prior to diagnosis was 6.3 months (range, less than 1 week to 36 months). Characteristics of the cohort and etiologies of CDI are reported in detail in Table 1. Etiologies included CNS malformations in 17 patients (48.5 %), intracranial masses in 13 patients (37.2 %), and Langerhans cell histiocytosis (LCH) in 3 patients (8.6 %). The underlying cause of CDI remains unclear in 2 patients (5.7 %) but both had abnormal MRI findings. Their cases are discussed in detail later in the article. No patients had a family history of DI. Median duration of follow-up was 5 years (range, 2 months to 16 years).

**Table 1:** Patient demographics and etiologies of central diabetes insipidus

Demographics	
Total Number of Patients, n	35
Female patients, n	14
Average Age of CDI Diagnosis, months	57.6 (0.73–192)
Time from Symptom Onset to CDI Diagnosis, months	6.4 (0.03–36)
Duration of Follow Up, months	60 (2.4–192)
Etiology	n (%)
*Central Nervous System Malformation*	*17 (48.5 %)*
Septo-Optic Dysplasia	9 (25.7 %)
Holoprosencephaly	5 (14.2 %)
Pituitary Hypoplasia	2 (5.7 %)
Encephalocele	1 (2.9 %)
*Intracranial Mass*	*13 (37.2 %)*
Craniopharyngioma	6 (17.1 %)
Astrocytoma	3 (8.6 %)
Germinoma	3 (8.6 %)
Pituitary Adenoma	1 (2.9 %)
*Langerhans Cell Histiocytosis*	*3 (8.6 %)*
*Unknown*	*2 (5.7 %)*

All patients underwent MRI of the brain. Thirty-three patients had neuroimaging performed at NCCH, and reports from referring hospitals were available for the remaining 2 patients. All but one had abnormalities on MRI of the brain including CNS malformations, intracranial masses, pituitary gland or stalk abnormalities. The one patient with a normal MRI of the brain was diagnosed with LCH (biopsy-proven) after presenting with a posterior auricular mass. Of the 17 patients with CNS malformations on MRI, 9 (25.7 %) had septo-optic dysplasia (SOD), 5 (14.2 %) had holoprosencephaly, 2 (5.7 %) had isolated pituitary hypoplasia, and 1 (2.9 %) had a large encephalocele. MRI revealed intracranial masses in 13 patients (37.2 %), including craniopharyngioma (6 patients, 17.1 %), astrocytoma (3 patients, 8.6 %), germ cell tumor (3 patients, 8.6 %), and pituitary adenoma (1 patient, 2.9 %). Four patients had pituitary stalk thickening. Of these patients, 2 were diagnosed with LCH, 1 was diagnosed with a germ cell tumor, and 1 has not yet undergone biopsy to determine a definitive diagnosis. One patient had absence of the posterior pituitary bright spot alone, without other structural abnormalities on MRI.

Six of 35 patients required formal water deprivation testing for diagnosis of CDI. The remaining patients already fulfilled the diagnostic criteria. All patients who required water deprivation test had abnormal findings on MRI of the brain, and etiologies for CDI included LCH, ectopic pituitary gland, pituitary hypoplasia, SOD, astrocytoma, and pituitary adenoma. Two of 6 patients had ADH deficiency alone at the time of diagnosis while the remainder had concurrent hormone deficiencies at presentation.

Twenty-one patients (60 %) had CDI at their initial presentation and 10 patients (29 %) developed CDI as a result of neurosurgical management of intracranial masses (6 with craniopharyngioma, 2 with astrocytoma, 1 with germinoma, and 1 with adrenocorticoptropic hormone (ACTH) secreting pituitary adenoma). Four patients (2 with SOD and 2 with ectopic posterior pituitary glands) did not have CDI at presentation but developed it during the follow-up period. One of the patients with SOD was diagnosed with thyrotropin (TSH), ACTH, and growth hormone (GH) deficiencies at birth but did not develop symptoms of CDI until 2 months of age. The second patient was diagnosed with SOD at 4 months of age but did not develop CDI until 12 years of age. One patient with an ectopic pituitary gland was diagnosed with panhypopituitarism except CDI at 2 months of age and developed CDI 1 year later. The other patient with an ectopic pituitary gland presented with GH deficiency and delayed puberty at 12 years of age and was diagnosed with CDI at 15 years of age.

GH deficiency was the most common concurrent hormone deficiency at presentation in patients with CDI and occurred in 12 patients (34 %). TSH, ACTH, and gonadotropin deficiencies were present at the time of diagnosis in 23 %, 23 %, and 6 % of patients, respectively. TSH deficiency was subsequently diagnosed in an additional 40 % of patients during follow-up. ACTH deficiency and gonadotropin deficiencies were later diagnosed in 37 % and 14 % of patients, respectively (Figure 1). Eighteen patients (51 %) were prepubertal in age so that the presence of gonadotropin deficiencies could not be assessed accurately. All 3 patients with LCH had CDI at presentation, and 1 developed subsequent hormone deficiencies as a result of disease progression and neurosurgery for diagnosis. Of the 5 patients with CDI as a result of holoprosencephaly, only 1 developed an additional hormone deficiency over time. The development of additional anterior hormone deficiencies occurred over time in 44 % of patients with SOD. Time from diagnosis of SOD to onset of subsequent hormone deficiencies ranged from months to years, with one patient having a normal pituitary evaluation at 4 months of age who did not develop subsequent hormone deficiencies until 12 years of age (see Table 2).

**Table 2:** Timing of development of pituitary hormone deficiencies in patients with cns malformations

Etiology	Age at diagnosis of CNS abnormality	Time from diagnosis of CNS abnormality to diagnosis of hormone deficiencies
Ectopic Pituitary	2 months	ACTH, GH, TSH, at presentation ADH, 1 year
Holoprosencephaly	3 months	ADH, at presentation TSH, 2 years
Pituitary Hypoplasia	13 years	GH, at presentation TSH, 9 months ADH, 2 years FSH, LH, 3 years
Septo-Optic Dysplasia	2 days	ACTH, GH, TSH, at presentation ADH, 2 months
Septo-Optic Dysplasia	4 months	GH,12 years ACTH, ADH, TSH, 13 years
Septo-Optic Dysplasia	8 months	ADH, GH, at presentation TSH, 1 year
Septo-Optic Dysplasia	22 months	ADH, GH, at presentation ACTH, 2 years

There were 2 patients with CDI of unclear etiology. The first patient has been followed at NCCH for 6 years. He has undergone MRI of the brain on an annual basis which has demonstrated a stable 4 mm focus of hypoenhancement within his right pituitary gland which may represent a Rathke’s cleft cyst or adenoma; however, he continues to follow-up with oncology given the possibility of LCH or germ cell tumor. He has had a total of two osseous surveys (2 years apart) that have failed to demonstrate evidence of LCH. He has undergone pituitary hormone screening every 6 to 12 months without development of subsequent hormone deficiencies. The other patient with CDI of unknown etiology is 13 year old female who presented for evaluation secondary amenorrhea, rapid weight gain, polydipsia and polyuria. She has been followed in our clinic for nearly 1 year, and her evaluation is significant for evidence of pituitary stalk thickening with absence of the posterior pituitary bright spot on MRI as well as TSH, GH, and gonadotropin deficiencies. A skeletal survey was normal. Neurosurgery has not yet agreed to obtain a biopsy for definitive diagnosis.

CDI in 31 patients was successfully managed with DDAVP. Chlorothiazide was used in 3 patients during infancy. One patient was managed with oral free water intake alone per the family’s preference. One patient with an astrocytoma had resolution of CDI after four years as a result of surgical intervention and chemotherapy.

## Discussion

In our series of pediatric patients with CDI, nearly all patients (97 %) had abnormalities on MRI of the brain, with CNS malformations and intracranial masses being the most common findings. Our findings are consistent with a recent study by Werny at al [4] that reports a higher prevalence of CNS malformations in patients with CDI than previously reported, as well as fewer idiopathic cases. We identified tumors or LCH in nearly half (46 %) of our patients with CDI, which is similar to the rate of tumors and infiltrative processes (56 %) found by Werny et al [4]. We did not identify any cases of familial/genetic, autoimmune, or idiopathic CDI, which is inconsistent with other previous studies citing familial causes in 7 % [4] and 6 % [5] and idiopathic etiology in 12 % [4] and 52 % [5]. These findings are likely a result of our small sample size. Despite traumatic causes of CDI being reported in 3 % of cases overall [5], we did not identify any cases. This is likely due to the fact that patients may have had either nonsurvivable brain injuries or transient CDI, and we included only patients followed in our outpatient clinic. Also congruent with the Werny study, our patients with pituitary stalk thickening were likely to be diagnosed with LCH or germ cell tumor [4-6]. Given the high likelihood of identifying an underlying etiology, it is of utmost importance to obtain neuroimaging promptly in patients diagnosed with CDI. In our cohort of patients with CDI, there was a high incidence of concurrent anterior pituitary hormone deficiencies. Hormone insufficiencies evolved over time in many of our patients with CNS malformations, particularly in those with SOD, or occurred abruptly as a result of neurosurgical intervention in patients with intracranial masses. Multiple pituitary hormone insufficiencies were identified at the time of diagnosis in 89 % of patients with SOD. Forty-four percent of patients with SOD developed additional hormone deficiencies anywhere from 2 months to 13 years after initial diagnosis [7], highlighting the importance of regular screening. This is in contrast to our patients with holoprosencephaly who were less likely to demonstrate evolution of anterior pituitary hormone deficiencies over time. These patients, who may have abnormal osmoreceptor function (previously termed “essential hypernatremia”), as a result of hypothalamic, rather than pituitary dysfunction [8, 9], may warrant less diligent hormonal screening evaluations over time compared to those with SOD; however, larger studies are needed to identify appropriate time intervals for repeat screening. The requirement for water deprivation testing to achieve the diagnosis of CDI was not associated with a specific underlying etiology or with the presence or absence of concurrent hormone deficiencies in our cohort.

CDI was managed successfully with DDAVP in the majority of patients in our cohort; however, patients in the neonatal period present a particular therapeutic challenge [8]. In our experience, DDAVP titration was problematic in such young patients and often resulted in wide fluctuations in sodium levels, including severe hyponatremia that occasionally resulted in seizures. In agreement with previous reports, we have been able to maintain acceptable sodium concentrations using chlorothiazide and low solute formula in infants up to 6 months of age, after which DDAVP doses may be titrated more easily [10, 11].

There are some limitations to our study that should be considered. First, the retrospective nature of our study results in inter-patient variability in the diagnosis of pituitary hormone deficiencies (random laboratory values versus provocative testing) and evaluation of hormone deficiencies over time. Furthermore, diagnostic laboratory data was limited in patients that had begun hormone replacement therapy prior to referral to our clinic. It is also likely that the incidence of gonadotropin deficiency is underreported given that many of our patients are too young to manifest symptoms of delayed puberty. Additionally, variability in MRI interpretation may result in under or over reporting of pituitary abnormalities. Finally, our population may not be representative of all children with CDI because we included only patients followed in the outpatient setting, excluding those with nonsurvivable CNS insults or transient CDI.

## Conclusions

Most children with CDI have abnormal findings on MRI of the brain, with intracranial masses and CNS malformations being most common. Therefore, the diagnosis of idiopathic CDI should be made with extreme caution in children, and only after extensive evaluation, including MRI of the brain, has been obtained. GH deficiency was the most common concurrent hormone deficiency at presentation in patients with CDI. TSH and ACTH deficiencies were more commonly diagnosed during follow-up evaluation; however, the development of additional hormone deficiencies in patients who did not undergo surgical intervention remains unpredictable, especially in those with SOD. Continued screening for endocrine dysfunction is warranted though further studies are needed to determine the most appropriate screening interval.

## Abbreviations

ACTH: Adrenocorticotropic hormone
CDI: Central diabetes insipidus
CNS: Central nervous system
DDAVP: Desmopressin
GH: Growth hormone
LCH: Langerhans cell histiocytosis
MRI: Magnetic resonance imaging
NCCH: North Carolina children’s hospital
SOD: Septo-optic dysplasia
TSH: Thyroid-stimulating hormone
